# Biomimetic Liver Lobules from Multi‐Compartmental Microfluidics

**DOI:** 10.1002/advs.202406573

**Published:** 2024-09-19

**Authors:** Danqing Huang, Zhuhao Wu, Ji Wang, Jinglin Wang, Yuanjin Zhao

**Affiliations:** ^1^ Department of Rheumatology and Immunology Nanjing Drum Tower Hospital Affiliated Hospital of Medical School Nanjing University Nanjing 210008 China; ^2^ Division of Hepatobiliary and Transplantation Surgery Department of General Surgery Nanjing Drum Tower Hospital Affiliated Hospital of Medical School Nanjing University Nanjing 210008 China; ^3^ Shenzhen Research Institute Southeast University Shenzhen 518071 China; ^4^ Institute of Organoids on Chips Translational Research Henan Academy of Sciences Zhengzhou 450009 China

**Keywords:** artificial liver, biomimetic, hepatic lobule, microfluidics, tissue engineering

## Abstract

Engineered liver lobule is highly practical in hepatic disease treatment, while constructing a 3D biomimetic lobule with a heterogeneous architecture on a large scale is challenging. Here, inspired by the natural architectural construction of hepatic lobules, biomimetic hepatic lobules are proposed with coaxially through‐pores for nutrient exchange via microfluidic technology. This multi‐channel microfluidic chip is made by parallelly installing capillaries. Sodium alginate (Alg) is pumped through its central channel, while Ca^2+^‐loaded gelatin methacrylate (GelMA) solutions encapsulating hepatocytes, mesenchymal stem cells, and endothelia cells are pumped through surrounding channels, respectively. The rapid gelation of Alg and Ca^2+^ brings about an in situ formation of Alg fiber, with heterogeneous multi‐cell‐laden GelMA microcarriers forming around it. The peeled‐off microcarriers each featured with a coaxially through pore, simulating the cord‐like structure of hepatic lobule and facilitating nutrients exchange. Meanwhile, the spatially anisotropic arrangement of cells highly simulates the hepatic architecture. It is demonstrated that by transplanting these biomimetic microparticles into liver in situ, the failed liver in rat shows increased regeneration and decreased necrosis. These results indicated that the microfluidic multi‐compartmental microcarriers provide a new strategy to engineer 3D artificial livers for clinical translation.

## Graphical abstract

Inspired by the natural architectural construction of hepatic lobules, biomimetic hepatic lobules are proposed with coaxially through‐pores for nutrient exchange via microfluidic technology aiming to engineer 3D artificial livers for clinical translation.

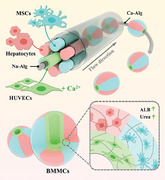

## 1 Introduction

As a promising therapeutic avenue, liver tissue engineering has advantages in liver disease treatment.^[^ ^1^, ^2^, ^3^, ^4^, ^5^ ^]^ Generally, hepatic lobule is the basic and functional unit of liver, composing hepatocytes, fibroblasts, Kupffer cells, endothelia cells, etc.^[^ ^6^, ^7^ ^]^ Nowadays, numerous technologies, such as micropatterning, scaffold reperfusion, bioprinting, and so on, have been developed to replicate the cell composition and arrangement of hepatic lobules under the condition of multi‐cell coculture.^[^ ^8^, ^9^, ^10^, ^11^, ^12^, ^13^ ^]^ Among them, 2D technologies like micropatterning can realize the homotypic and heterotypic interactions between hepatocytes and other cells, contributing to cellular polarization and enhanced hepatic‐like functions.^[^ ^14^, ^15^, ^16^ ^]^ In contrast, 3D bioprinting technology has shown great advantages in building artificial livers with biological functions and stable mechanical properties.^[^ ^5^, ^17^, ^18^, ^19^ ^]^ However, it might be problematic for 3D bioprinting to construct multi‐component independent bio‐microunits. Therefore, engineering technology for micro‐scale biomimetic hepatic lobules establishment remains to be explored.

**Figure 1:**
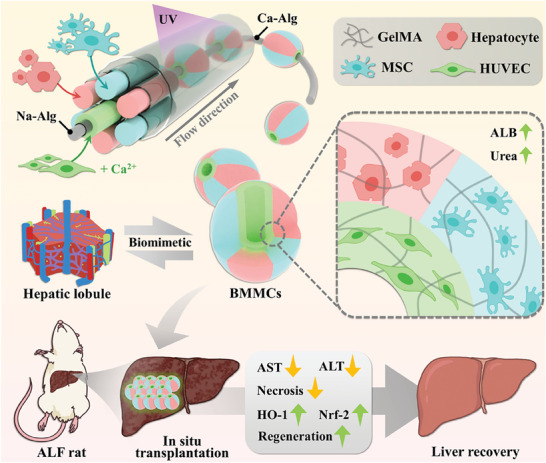
Schematic illustration of the biomimetic multi‐compartment microparticles via microfluidics for liver failure treatment.

Here, inspiration from the natural structure of hepatic lobules, we have developed a novel multi‐compartmental microfluidic technology for the generation of biomimetic hepatic lobules with coaxially through‐pores for nutrients exchange, as illustrated in **Figure** **1**. Microfluidic technology has significant advantages in fabricating microcarriers with precise structures and tailored compositions.^[^ ^20^, ^21^, ^22^ ^]^ Compared with other tissue engineering approaches, microfluidic methods can encapsulate cells in a gentle and maneuverable way. Moreover, ascribing to the glass capillary‐based microfluidic chips, the micro‐scale architectures of the microcarriers can be highly customizable and the fabrication process is low‐cost and high‐throughput.^[^ ^23^, ^24^, ^25^, ^26^ ^]^ Nowadays, studies have proposed various biomimetic microcarriers with core‐shell, Janus, multi‐compartmental structures via microfluidic technology.^[^ ^27^, ^28^, ^29^ ^]^ However, to ensure nutrients supply, size and cell content of these microcarriers are severely restricted. Additionally, these microcarriers inadequately replicate the complex structures of the liver lobules well, resulting in insufficient cell‐cell and cell‐extracellular matrix interactions, limiting their hepatic‐like functions. Therefore, new biomimetic microfluidic microcarriers with effective pathways for nutrients exchange are highly anticipated for hepatic tissue engineering.

In this paper, we present the desired multi‐compartmental microcarriers with a coaxially through pore imitating the micro‐architecture and construction of hepatic lobule via microfluidic technology. Since hepatocytes are arranged in a cord‐like architecture around a vein and are separated by sinusoids, we designed and constructed a multi‐compartmental microfluidics with the same cross‐section architecture using glass capillaries. Attributing to the rapid ionic crosslinking gelation of sodium alginate (Na‐Alg) and calcium ions in the microfluidics, a calcium alginate (Ca‐Alg) fiber formed in situ surrounded with heterogeneous gelatin methacrylate (GelMA) emulsions. With the photocuring of ultraviolet (UV) light, multi‐compartmental GelMA microparticles formed and can be peeled off from the Ca‐Alg fiber, resulting in heterogeneous microparticles with a coaxially through pore. Additionally, by loading hepatocytes, mesenchymal stem cells (MSCs), and human umbilical vein endothelial cells (HUVECs) into different microfluidic channels, respectively, we achieved biomimetic multi‐compartmental microcarriers (BMMCs) with hepatic lobule‐imitating morphology and functionality. We have demonstrated that in rats with acute liver failure (ALF), in situ transplantation of these biomimetic microparticles can significantly reduce hepatocytes necrosis and improve liver regeneration. Therefore, our microfluidic multi‐compartmental microparticles provide a new strategy for 3D engineered liver in clinical translation.

## 2 Results and Discussion

**Figure 2:**
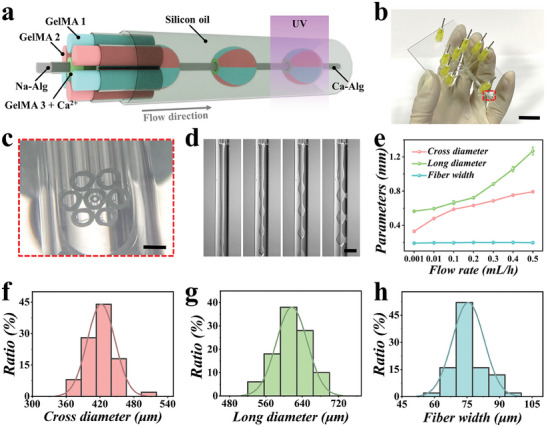
Microfluidic fabrication of multi‐compartment microparticles. a) Schematic illustration of the multi‐compartmental microfluidics. b) Photograph of the multi‐compartment microfluidic chip. c) Stereomicroscopic image of the orifice. d) Real‐time images of the generation process of microfibers and coating droplets. e) Relationship between the cross diameter, long diameter, fiber width, and GelMA flow rate (n = 5). f–h) Diameter distribution of the long diameter (f), cross diameter (g), and fiber width (h) under 0.01 mL h^−1^ GelMA flow rate, 0.05 mL h^−1^ Alg flow rate, and 1 mL h^−1^ silicon oil flow rate. Scale bars are 1 cm in (b), 250 µm in (c), and 1 mm in (d).

In a typical experiment, the multi‐compartmental microparticles were fabricated using a multi‐channel microfluidic chip, as illustrated in **Figure** **2**a. The microfluidic chip was designed and fabricated using capillaries, glass slide, and needles with plain ends (Figure S1, Supporting Information). First, seven capillaries with 580 µm inner diameter were assembled in parallel using photocuring glue to form an annular capillary array. Then, we tapered the seven‐channel annular capillary array and assembled it with a collection capillary with 1.2 mm inner diameter coaxially on a glass slide (Figure 2b; Figure S2, Supporting Information). To facilitate different fluid entry into different channels, seven tapered and bent capillaries were inserted into the capillary array respectively. Furthermore, a tapered capillary was inserted into the central capillary of the seven‐channel annular capillary array coaxially (Figure 2c). Finally, we applied eight needles to seal the entrance of every capillary, and a handmade multi‐channel microfluidic chip was achieved. To verify its feasibility for fabricating multi‐compartmental microparticles, we first pumped GelMA pregel solution dyed with red and blue fluorescence into the surrounding four or six channels. As shown in Figure S3 (Supporting Information), microparticles with two, four, and six well‐defined compartments can be achieved. Based on these results, we added a pump of GelMA pregel solution dyed with green fluorescence into the central channel of the capillary array. As illustrated in Figure S4 (Supporting Information), microparticles with green fluorescent core and two, four, and six well‐defined compartmental shell can be achieved.

Encouraged by the results, we additionally pumped Na‐Alg solution into the inner channel and added calcium ions into the GelMA pregel solution. Ascribing to the quick diffusion of calcium ions, Ca‐Alg hydrogel fiber formed when the multi‐phase fluid advancing through the collection capillary. Meanwhile, due to the interaction between the interface tension of the GelMA solutions and the shear force of the silicon oil, the multi‐compartmental GelMA pregel solution was emulsified and formed into droplets coating the Ca‐Alg fiber (Figure 2d). Notably, by adjusting the flow rate of the GelMA pregel solutions, we can achieve fibers with different diameters and microparticles with different long and cross diameters (Figure 2e; Figure S5, Supporting Information). Moreover, by measuring 50 microparticles fabricated under different microfluidic flow rates, it can be concluded that these microparticles showed uniform morphology and batch‐to‐batch similarity (Figure 2f–h).

**Figure 3:**
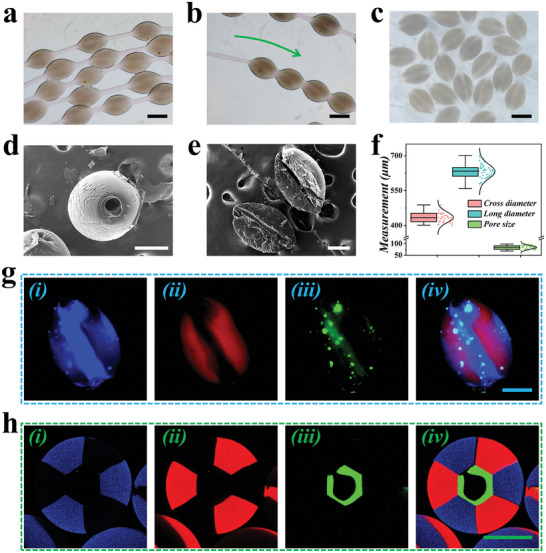
Characterization of the multi‐compartmental microparticles with coaxially through pore. a) Optical microscopic image of the multi‐compartmental microparticles coating microfibers. b) Optical microscopy showed the free movements of the multi‐compartmental microparticles along the microfiber. c) Optical microscopic image of the multi‐compartmental microparticles with coaxially through pore after sliding off the fibers. d,e) SEM images showed the coaxially through pore within the microparticle. f) Size distribution of the cross diameter, long diameter, and pore size (n = 40). g,h) Fluorescence microscope images (g) and CLSM images (h) of the multi‐compartmental microparticles. Scale bars are 400 µm in (a–c), and 200 µm in (d,e,g,h).

By photocuring GelMA with ultra‐violet lights, polymerized GelMA microparticles strung along the Ca‐Alg fiber can be achieved (**Figure** **3**a). Since the polymerized GelMA and the Ca‐Alg fiber were independent, the GelMA microparticles can move through the fiber freely (Figure 3b). After peeling the GelMA microparticles off the fiber, multi‐compartmental microparticles with a coaxially macropore can be achieved (Figure 3c; Figure S6, Supporting Information). As shown in the SEM image, the macropore can be observed coaxially located in the microparticle, which might be facilitating the exchange between the microparticle and the surrounding environment (Figure 3d,e). As illustrated in Figure 3f, the microparticles showed uniform morphology (Figure S7, Supporting Information). It can be observed from fluorescent microscopy that the multi‐compartmental microparticle featured with a coaxially located green fluorescent core surrounded by red and blue fluorescence compartments (Figure 3g). Moreover, we applied confocal laser scanning microscopy (CLSM) to further observe the cross‐sectional structure of the microparticles. As shown in Figure 3h and Figure S8 (Supporting Information), the six components around the microsphere were evenly distributed and clearly demarcated, meanwhile, the green fluorescent part in the middle appeared hollow cord‐like structure, which is highly similar to the architecture of hepatic lobules.

**Figure 4:**
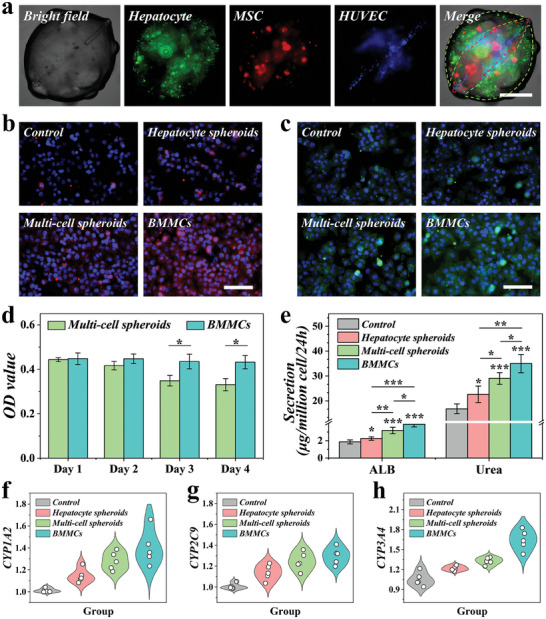
Establishment and functional evaluation of BMMCs. a) Bright field and fluorescent images of BMMCs loading hepatocytes (green fluorescence), MSCs (red fluorescence), and HUVECs (blue fluorescence). The green dotted lines represent the approximate range of hepatocytes' location, and the red dotted line represents the approximate range of MSCs' location. b,c) Immunofluorescence staining of ALB (b) and CYX3A4 (c). d) Cell counting kit‐8 analysis of cells cultured in microspheres with different structures (n = 5). (e) ALB secretion and urea synthesis analysis from different groups (n = 5). f–h) Q‐PCR analysis results of f) CYP1A2, g) CYP2C9, and h) CYP3A4 gene expression from different groups (n = 5). Scale bars are 200 µm in (a) and 100 µm in (b,c). The statistical significance is indicated by ^*^ *p* < 0.05, ^**^ *p* < 0.01, and ^***^ *p* < 0.001.

Compared with 2D culture, multiple cells co‐cultured in arranged 3D architectures would enhance cell functionalities. For this purpose, we dispersed hepatocytes, MSCs, and HUVECs into GelMA solutions, respectively, and pumped these pregel solutions into the multi‐channel microfluidic device. As illustrated in **Figure** **4**a, hepatocytes and MSCs were interlacedly distributed around the periphery of the microsphere, with HUVECs landed along the coaxially cord‐like structure. Ascribed to the coaxially pore through the microsphere, nutrient and waste exchange can be facilitated, especially for the cells located in the center position. GelMA hydrogel has long been applied for biomedical application due to its excellent biocompatibility.^[^ ^30^ ^]^ Cells encapsulated in GelMA hydrogel showed ideal viabilities. By comparing the viabilities of cells encapsulated in GelMA microspheres with and without coaxially through pore, we found that our BMMCs are more conducive to the maintenance of cell activity and long‐term culture (Figure 4d; Figure S9, Supporting Information).

Then, we investigated whether co‐culture of multiple cells and special cell spatial arrangement could improve their hepatic‐like functions. Hepatocytes cultured in plate were set as control. We also fabricated hepatocyte‐encapsulated spheroids and multi‐cell encapsulated sold spheroids. Then the co‐culture models  were divided into 4 groups, namely Control group, Hepatocyte spheroids group, Multi‐cell spheroids group, and BMMCs group. After co‐culturation, it was found that the hepatocytes co‐cultured with hepatocyte‐encapsulated spheroids, multi‐cell encapsulated spheroids and BMMCs showed significantly enhanced albumin (ALB) secretion and urea synthesis, indicating their advanced hepatic functions (Figure 4b,e; Figure S10, Supporting Information). Notably, the hepatocytes in BMMCs group showed improved functions compared with the other two groups. Additionally, it was evaluated through immunofluorescence staining and real‐time quantitative polymerase chain reaction (qPCR) that the expressions of drug metabolism‐related genes, including CYP1A2, CYP2C9, and CYP3A4, were significantly enhanced in BMMCs group, implying that the BMMCs can effectively exert hepatic‐like functions (Figure 4c,f–h; Figure S11, Supporting Information).

**Figure 5:**
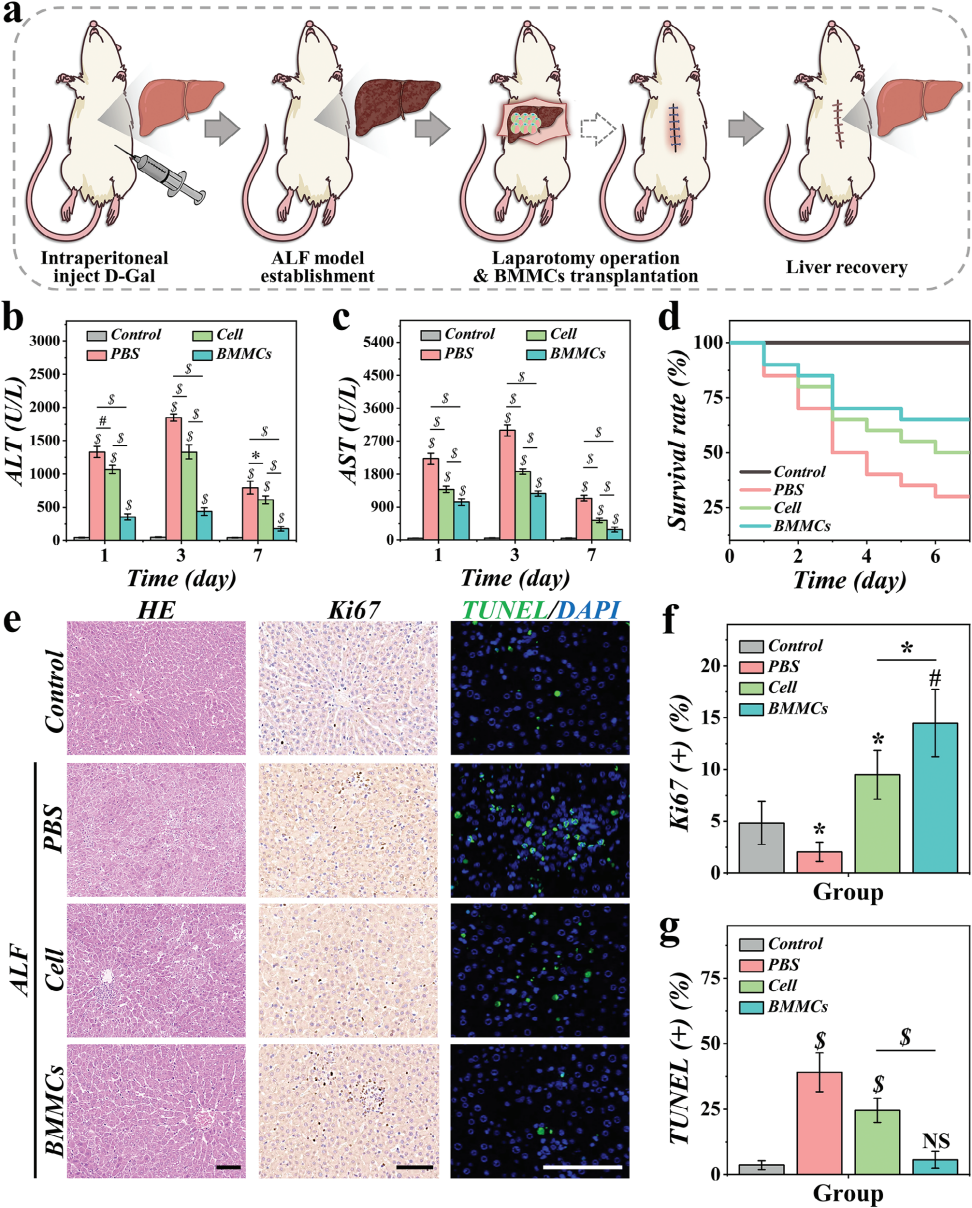
Biomimetic multi‐compartmental microparticles for ALF rat treatment. a) Schematic illustration of the animal experiment process. b,c) The ALT and AST levels of rats from different groups during treatment (n = 5). d) The survival rate of rats from control, PBS, Cell, and BMMCs group (n = 20). e) The HE, Ki67, and TUNEL staining of liver samples from different groups. f,g) Statistical analysis of Ki67 (f) and TUNEL (g) positive rates (n = 5). The statistical significance is indicated by NS: not significant, *p < 0.05, #*p* < 0.01, and $*p* < 0.001. Scale bars are 100 µm in (e).

Encouraged by the positive results from in vitro experiments, we applied BMMCs to treat rats with ALF. After injecting D‐galactosamine (D‐Gal) intraperitoneally to establish ALF rat models, these rats were randomized into three groups, namely, PBS group, Cell group, and BMMCs group. Normal rats were set as control. For the experimental groups, PBS, hepatocytes suspension, and BMMCs were applied to rats in PBS group, Cell group, and BMMCs group, respectively (**Figure** **5**a; Figure S12, Supporting Information). During the 7‐day treatment, blood samples were extracted, and the ALT and AST levels were examined at day1, 3, and 7. The ALF rats treated with PBS showed rapidly increased and persistently high levels of ALT and AST, while those treated with hepatocytes and BMMCs showed decreased ALT and AST (Figure 5b,c). Notably, it can be concluded from the results that the liver failure of rats from the BMMCs group was efficiently controlled, and that their liver function recovered significantly faster than the other two groups. Consistent with the liver function indicators, the survival rate of the BMMCs group was significantly better (Figure 5d). Additionally, it can be observed from the Hematoxylin‐eosin (HE) staining results that the liver of the rats from the BMMCs group showed reduced vacuolization, decreased inflamed areas, and significantly repaired damages (Figure 5e). By conducting Ki67 staining and measuring the Ki67 positive rates, we found that the BMMCs treatment can efficiently improve the proliferative activity of liver cells (Figure 5e,f). We further evaluated the cell apoptosis in the liver of rats from different groups through terminal deoxynucleotidyl transferase nick end labeling (TUNEL) assay. Results showed that after 7‐day treatment, the apoptosis rate of liver cells in the BMMCs group was significantly reduced (Figure 5e,g; Figure S13, Supporting Information). These results indicated that BMMCs can effectively ameliorate liver injury and promote liver repair.

**Figure 6:**
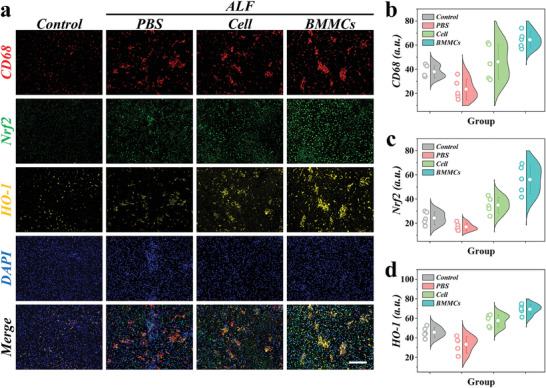
Immunohistochemical evaluation of the therapeutic efficacy of biomimetic microparticles. a) Representative CD68 (red fluorescence), Nrf2 (green fluorescence), HO‐1 (yellow fluorescence), DAPI (blue fluorescence), and merge fluorescent images of liver samples from different groups. b–d) Fluorescence quantitative analyses of b) CD68, c) Nrf2, and d) HO‐1. Scale bar is 200 µm in (a).

Due to the inevitable presence of inflammatory damage and oxidative stress responses in ALF, a cytokine storm can exacerbate liver damage, creating a vicious cycle. To further explore the effect of BMMCs on promoting damaged liver repair, CD68, nuclear factor E2 related factor (Nrf2), and Heme oxygenase‐1 (HO‐1) immunofluorescence staining and relative quantitative statistics were performed in liver samples of rats from different groups. Compared with control and PBS group, the CD68 expression was elevated in BMMCs group, indicating the aggregation of macrophages and the enhancement of anti‐inflammatory capacity (**Figure** **6**a,b). It was reported that the activation of Nrf2/HO‐1 antioxidant pathway can promote liver recovery.^[^ ^31^ ^]^ The Nrf2 and HO‐1 gene expression was significantly increased in BMMCs group, suggesting the antioxidant capacity of ALF rats treated with BMMCs was enhanced (Figure 6a,c,d). These results indicated that the treatment of BMMC transplantation can ameliorate liver injury through anti‐inflammation and antioxidation.

## 3 Conclusion

In conclusion, inspired by the free movement of the beads on a string, we designed a multi‐channel microfluidic chip and fabricated liver‐mimetic multi‐compartmental microparticles with coaxially located macro pore. Consistent with the architecture of hepatic lobules, our microparticles featured with spatially anisotropic compartments and cord‐like hollow structure. Ascribing to the maneuverability of microfluidic technology, the fabricated microparticles showed customized morphology and fine‐tuned size. Compared with other biological fabrication methods, microfluidic allows high‐throughput production and batch‐to‐batch similarity, showing advantages for massive and wide application. By loading hepatocytes, MSCs, and HUVECs into different hydrogel compartments, the BMMCs showed enhanced hepatic‐specific functions. Moreover, due to the coaxially located macro pore through the BMMCs, nutrients exchanged was facilitated, which contributed to the cell viability encapsulated in the microparticles. The practicability of BMMCs was further verified by treating ALF rats. Through Ki67 and TUNEL immunostaining, it can be observed that the liver samples of BMMCs‐treated rats showed enhanced regeneration and decreased necrosis. Furthermore, the in situ transplantation of BMMCs significantly increased the expression of Nrf2 and HO‐1, suggesting the antioxidant capacity of the damaged liver was improved. Taken together, we proposed a novel liver engineering method using a customized multi‐channel microfluidic chip and realized structurally organized multi‐cell arrangement to simulate hepatic lobule architecture and functions. Therefore, our BMMCs provided a promising approach for liver reconstruction and ALF treatment.

## 4 Experimental Section

### Fabrication of Multi‐Channel Microfluidic Chip

Seven capillaries with inner diameter of 580 µm and same length were assembled in a hexagonal compact pile using photocuring glue. Then, the annular capillary array was heated and drawn using an alcohol lamp, followed by sanding into a total diameter of ≈ 800 µm. A collection capillary with inner diameter of 1.2 mm used to pump silicon oil was assembled coaxially with the tapered seven‐annual capillary on a glass slide. To facilitate the fluids flowing through the capillary channels, seven capillaries with inner diameter of 580 µm were bent and tapered using an alcohol lamp, followed by inserting into the capillaries of the assembled seven‐annual capillary. Furthermore, a tapered capillary with inner diameter of 580 µm was inserted into the central capillary coaxially. Finally, the entrances of eight channels and collection capillary were sealed with needles and epoxy resin, respectively.

### Fabrication of Multi‐Compartmental Microparticles

Na‐Alg solution pumping into inner capillary was prepared using 1.0 wt.% sodium alginate (medium viscosity). GelMA solution pumping into central capillary was prepared using 10.0 wt.% GelMA, 0.1 vol.% photoinitiator 2‐hydroxy‐2‐methylpropiophenone, 1.0 wt.% calcium chloride, and 0.01 vol.% green fluorescent nanoparticles. GelMA solutions pumping into outer capillaries were prepared using 10.0 wt.% GelMA, 0.1 vol.% photoinitiator 2‐hydroxy‐2‐methylpropiophenone, and 0.01 vol.% red or blue fluorescent nanoparticles. Silicon oil (100cs) was pumped through the collection capillary. UV light (365 nm, Ontario, Canada) was applied to irradiate the collection capillary. Syringe pumps (Harvard PHD 2000 series) were applied to regulate the flow rates of different solutions. During microfluidic process, the flow rate of GelMA solution was regulated to 0.001, 0.01, 0.1, 0.2, 0.3, 0.4, and 0.5 mL h^−1^, and the corresponding cross diameter, long diameter and fiber width were recorded.

### Fabrication of Biomimetic Microparticles

Consistent with the fabrication of multi‐compartmental microparticles, Na‐Alg and GelMA solutions were prepared using same formulation. Specially, GelMA solution pumping into central capillary was prepared with addition of HUVECs (2.5 × 10^5^ cells/mL), and GelMA solutions pumping into outer capillaries were prepared with addition of MSCs (2.5 × 10^5^ cells/mL) and hepatocytes (5 × 10^5^ cells/mL), respectively. The hepatocytes were induced from iPSCs through the previous protocol.^[^ ^32^ ^]^ After the polymerization and collection of the microparticles, these biomimetic microparticles were cultured for subsequent use.

### Function Assessment of the BMMCs

To evaluate the cell distribution of hepatocytes, MSCs, and HUVECs loaded in microparticles, these cells were dyed with green, red and blue before encapsulation and were observed under fluorescence microscopy. In vitro co‐culture models were randomized into Control group, Hepatocyte spheroids group, Multi‐cell spheroids group, and BMMCs group. Control group left untreated. Hepatocyte spheroids group, Multi‐cell spheroids group, and BMMCs group was co‐cultured with hepatocytes spheroids, multi‐cell spheroids (containing hepatocytes, MSCs, and HUVECs), and BMMCs fabricated via multi‐channel microfluidics. After 4‐day culturation, immunofluorescence staining of ALB and CYX3A4 was conducted. The ALB secretion and urea synthesis were measured using the ELISA Quantitation kit and Urea Nitrogen kit, respectively.

### Animal Experiments

Eighty Sprague−Dawley rats were randomized into Control group, PBS group, Cell group, and BMMCs group (20 rats each group). The establishment of ALF rat models were conducted according to the previous studies.^[^ ^9^, ^23^ ^]^ D‐Gal (0.6 g kg^−1^ rat weight) were intraperitoneal injected to rats in PBS group, Cell group, and BMMCs group to establish ALF models. For Control group, rats left untreated. For PBS, Cell and BMMCs group, rats received in situ injection of PBS, hepatocyte suspension (same hepatocyte quality of BMMCs group, 4 × 10^5^ hepatocytes approximately) and BMMCs (300 microparticles each rat, totaling 1 × 10^6^ cells approximately), respectively. All rats survived after laparotomy. In the next 7 days, the survival rate was recorded every day; ALT and AST level was measured on day 1, day 3, and day 7. After 7‐day treatment, livers of rats from different groups were harvested and conducted HE, Ki67, TUNEL, CD68, Nrf2, and HO‐1 staining.

## Conflict of Interest

The authors declare no conflict of interest.

## Author Contributions

Y.J.Z. conceived the idea and designed the experiment; D.Q.H. and Z.H.W. participated in the production and characterization of the multi‐compartmental microparticles; D.Q.H. and J.L.W. carried out the bio‐experiments and analyzed the data; D.Q.H drew all the figures and wrote the manuscript; J.L.W. and J.W. contributed to the scientific discussion.

## Supporting information



Supporting Information

## Data Availability

The data that support the findings of this study are available from the corresponding author upon reasonable request.
