# Supplementary material for: Biomimetic Liver Lobules from Multi‐Compartmental Microfluidics
Source: Adv Sci (Weinh). 2024 Sep 19;11(42):2406573. doi: 10.1002/advs.202406573 (PMC11558095; doi:10.1002/advs.202406573)
Supplement: Supplementary file 1 — Supporting Information [file ADVS-11-2406573-s001.docx]

Supporting Information

Biomimetic liver lobules from multi-compartment microfluidics

Danqing Huang, Zhuhao Wu, Ji Wang, Jinglin Wang*, Yuanjin Zhao*

**Materials and methods**

***Materials, cells and animals:*** Capillaries with 580 μm and 1.2 mm inner diameter were purchased from World Precision Instruments. Sodium alginate (Na-Alg) (medium viscosity), calcium chloride (CaCl_2_), gelatin, methacrylic acid, 2-hydroxy-2-methylpropiophenone were purchased from Sigma-Aldrich., USA. Methacrylate gelatin (GelMA) was synthesized using gelatin and methacrylic acid in laboratory. Silicone oil (100cs) was purchased from Shinetsu, Japan. Blue fluorescent nanoparticles F8805 (excitation/emission: 365/415 nm), Red fluorescent nanoparticles F8810 (excitation/emission: 580/605 nm), and green fluorescent nanoparticles F8811 (excitation/emission: 505/515 nm) were purchased from Thermo Fisher Scientific, USA. Fetal bovine serum (FBS), penicillin/streptomycin (P/S), Trypsin-EDTA solution, phosphate buffer saline (PBS) was purchased from Gibco. Cell Counting Kit-8 (CCK-8) was obtained from Dojindo. Human umbilical vein endothelial cells were obtained from the Cell Bank of the Chinese Academy of Sciences, Shanghai, China. The Sprague−Dawley rats were purchased from the Model Animals Research Center of Nanjing University. All animal experiments were strictly in accordance with guidelines set by the Animal Ethics Committee of Drum Tower Hospital affiliated to Medical School of Nanjing University (No.20230401).

**Figures**

Figure S1. Schematic illustration of the fabrication of multi-channel microfluidic chip.

Figure S2. Front and side photographs of the multi-compartment microfluidic chip.

Figure S3. Microparticles with two, four, and six compartments.

Figure S4. Microparticles with green fluorescent core and two, four, and six compartment-shell.

Figure S5. Scheme of measurement methods of long diameter, cross diameter, and fiber width.

Figure S6. Achievement of the multi-compartmental microparticle with a coaxially macropore.

Figure S7. Scheme of measurement methods of long diameter, cross diameter, and pore size.

Figure S8. The three-dimensional confocal image and the cross-sectional images of the multi-compartmental microparticles.

Figure S9. Calcein AM staining of the BMMCs.

Figure S10. Single channel fluorescence staining images of DAPI and ALB from different groups.

Figure S11. Single channel fluorescence staining images of DAPI and CYX3A4 from different groups.

Figure S12. Photograph of the animal experiment process.

Figure S13. Single channel fluorescence staining images of TUNEL and DAPI from different groups.


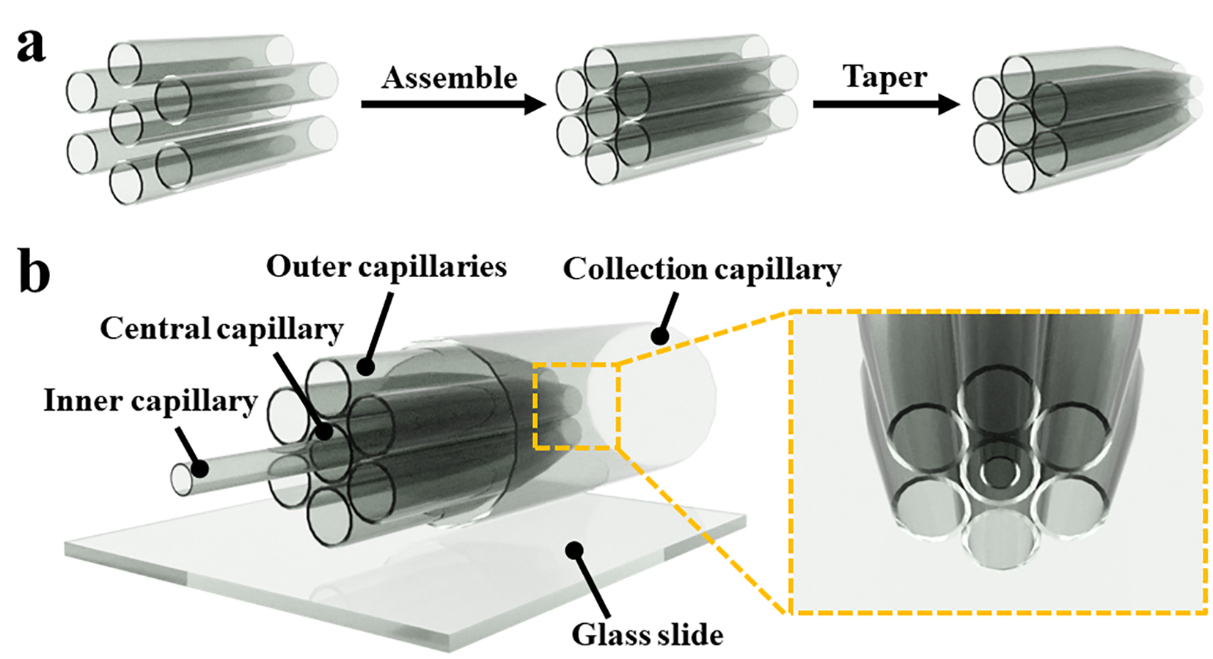


**Figure S1.** Schematic illustration of the fabrication of multi-channel microfluidic chip.


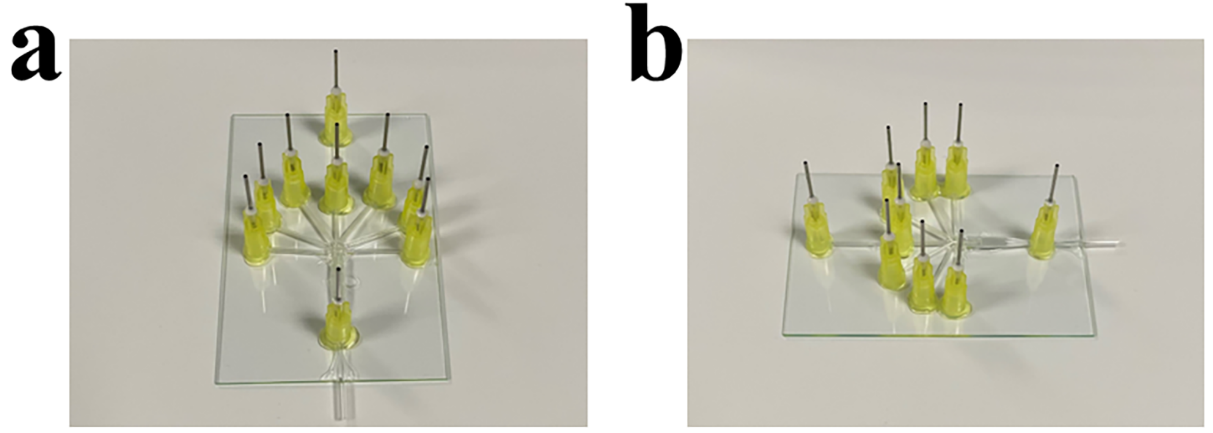


**Figure S2.** Front (a) and side (b) photographs of the multi-compartment microfluidic chip.


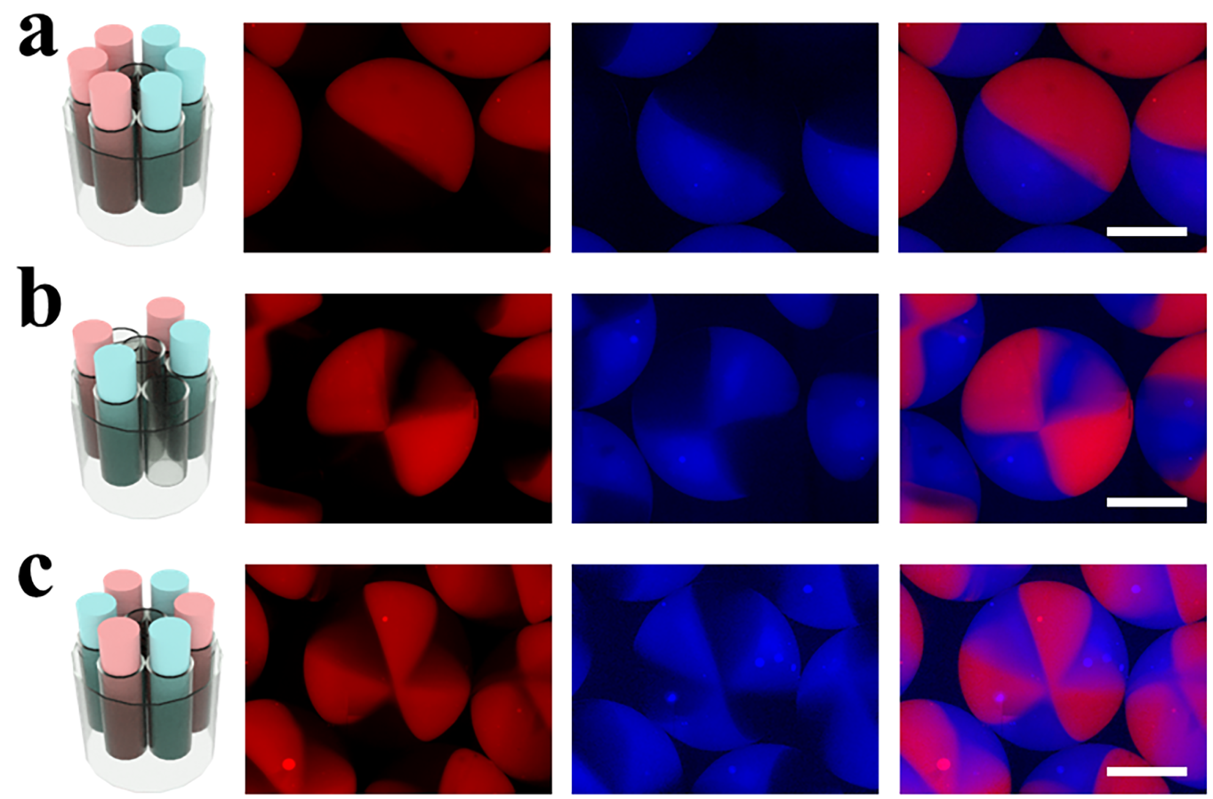


**Figure S3.** Microparticles with two (a), four (b), and six (c) compartments. Sale bars are 200 μm in (a-c).


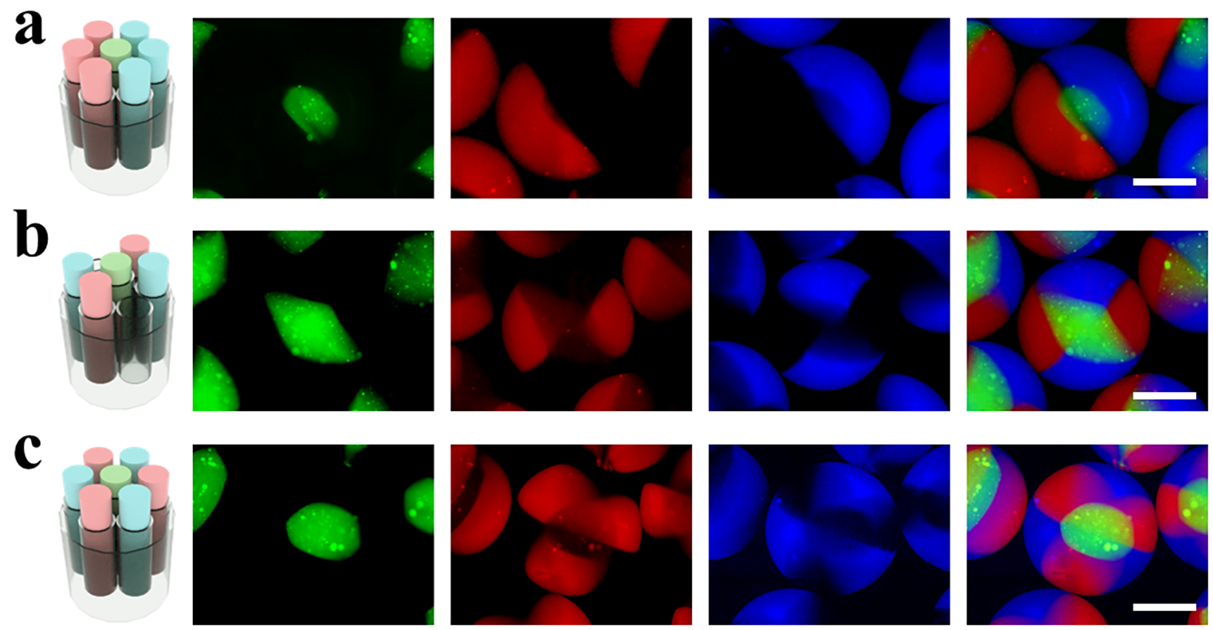


**Figure S4.** Microparticles with green fluorescent core and two (a), four (b), and six (c) compartment-shell. Sale bars are 200 μm in (a-c).


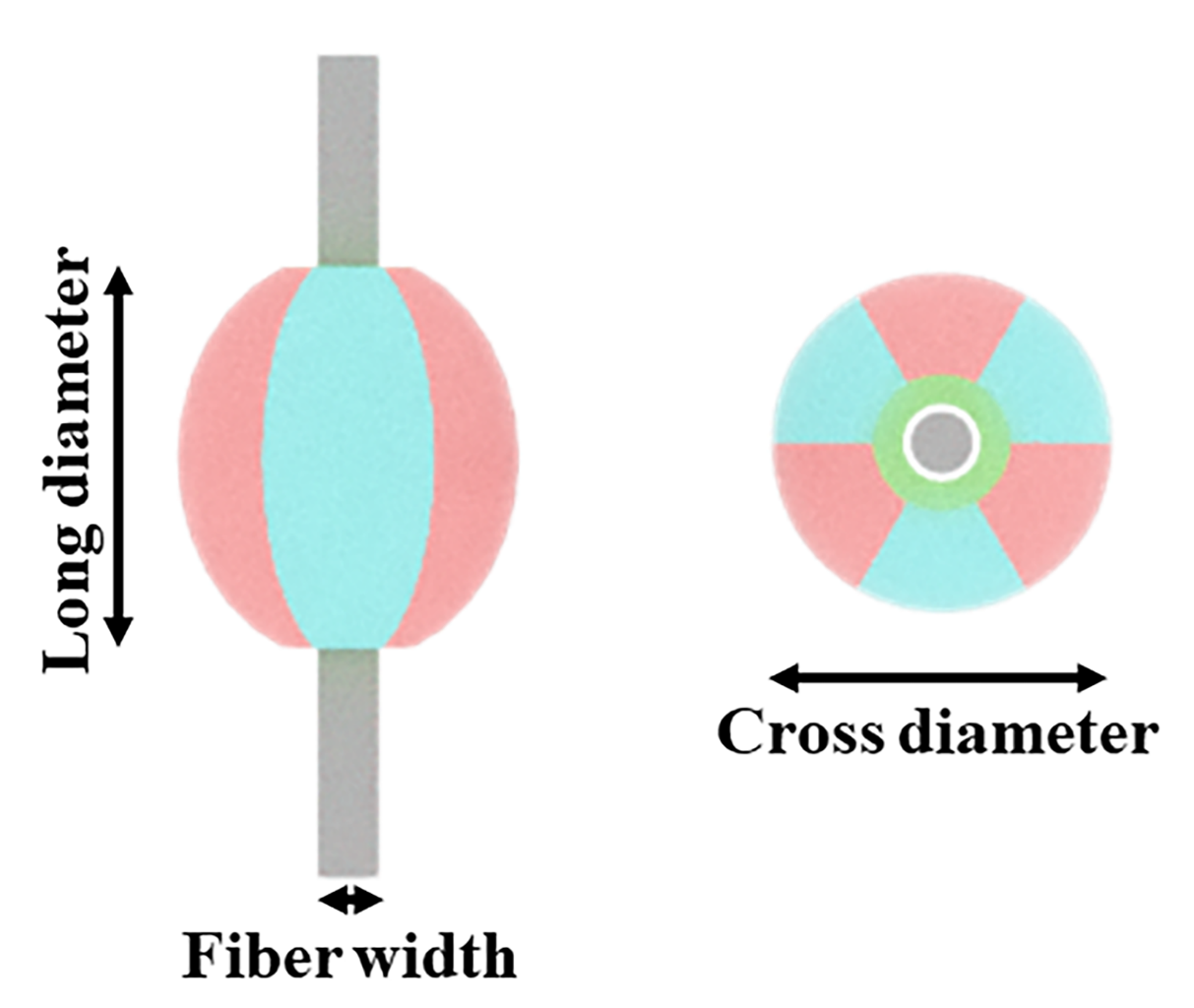


**Figure S5.** Scheme of measurement methods of long diameter, cross diameter, and fiber width.


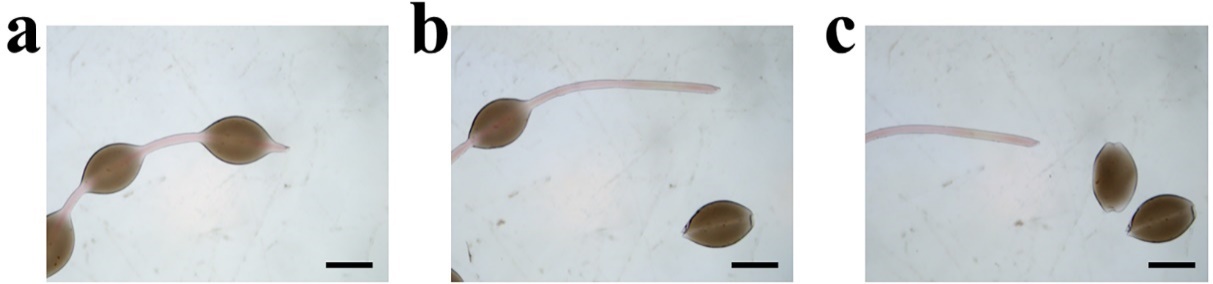


**Figure S6.** Achievement of the multi-compartmental microparticle with a coaxially macropore. (a) Microparticles on a fiber. (b) The first microparticle comes off the fiber. (c) The second microparticle comes off the fiber. Scale bars are 400 μm in (a-c).


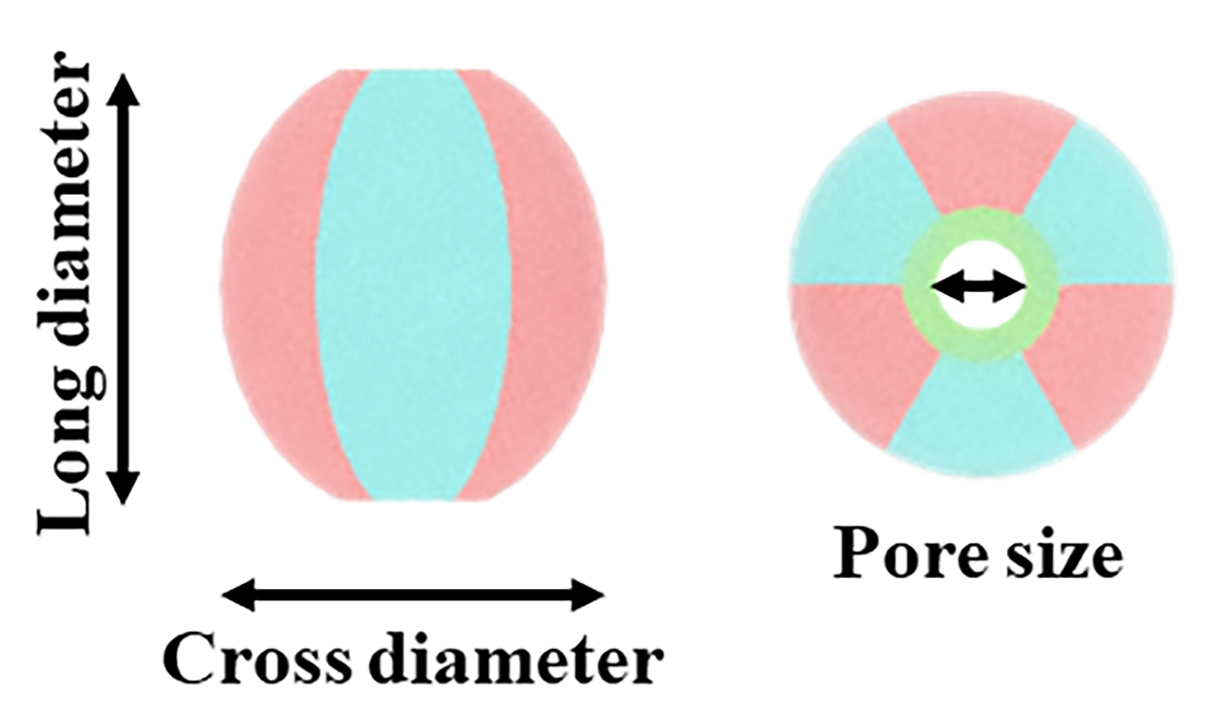


**Figure S7.** Scheme of measurement methods of long diameter, cross diameter, and pore size.


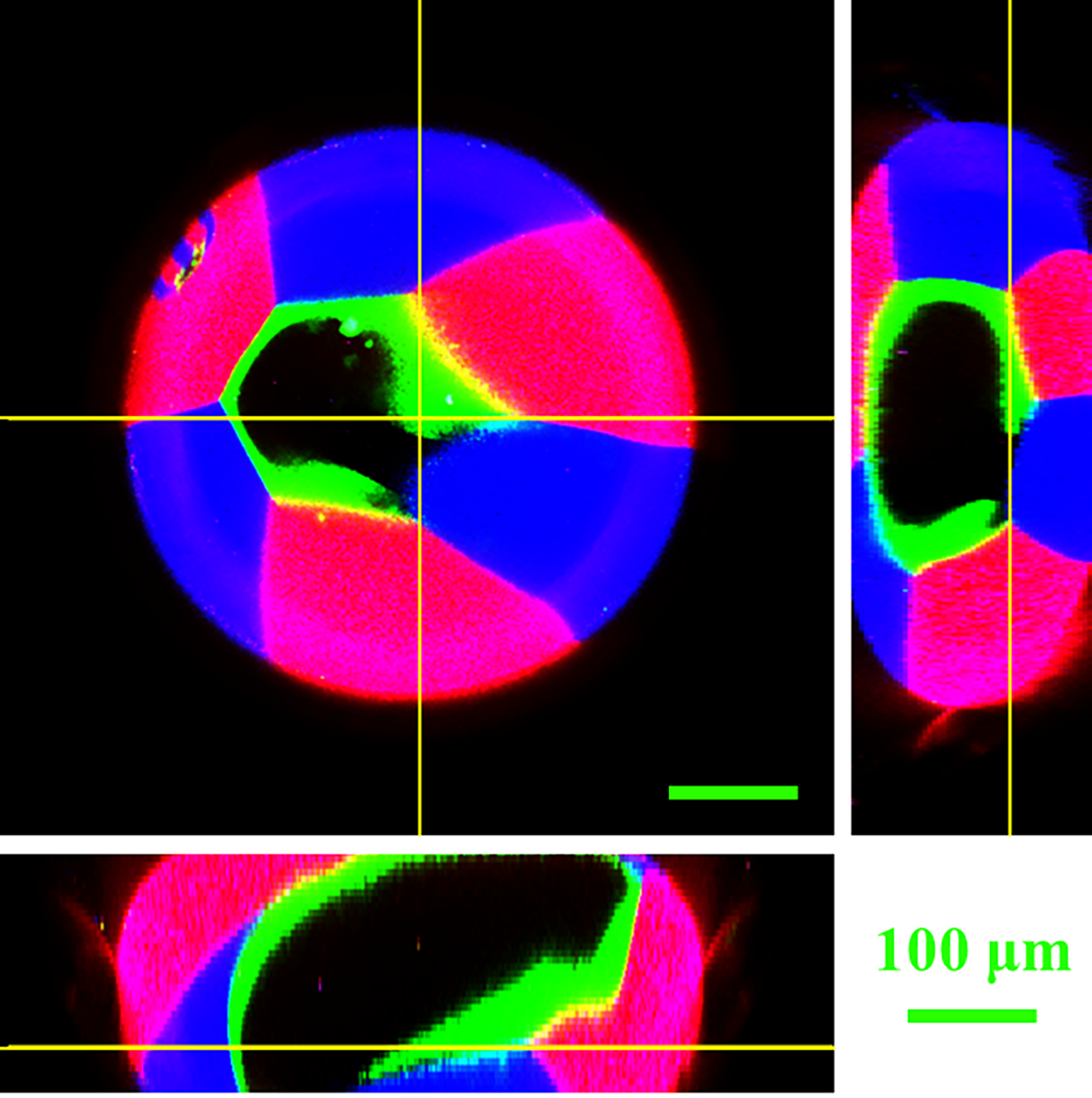


**Figure S8**. The three-dimensional confocal image and the cross-sectional images of the multi-compartmental microparticles. Scale bar is 100 μm.


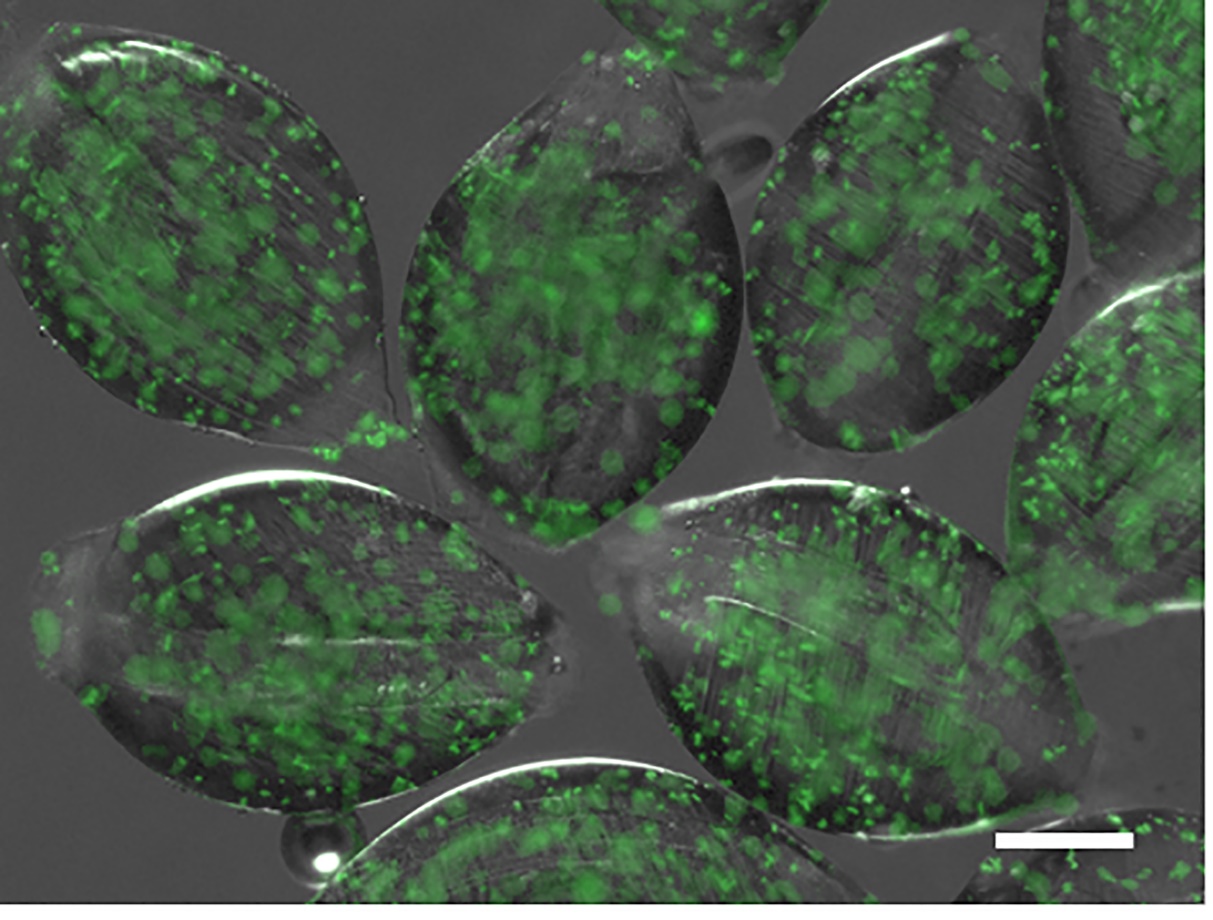


**Figure S9.** Calcein AM staining of the BMMCs. Sale bar is 200 μm.


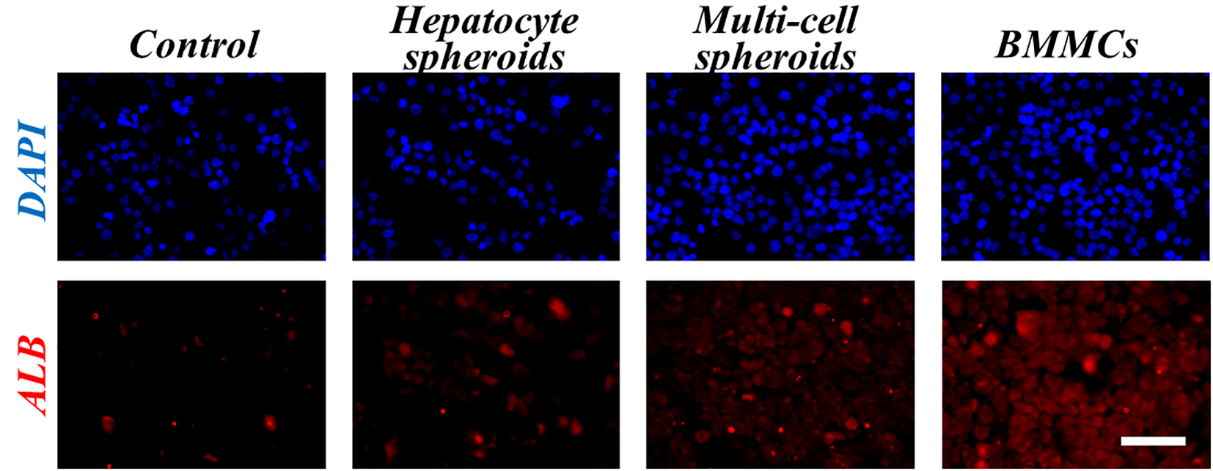


**Figure S10.** Single channel fluorescence staining images of DAPI and ALB from different groups. Scale bar is 100 μm.


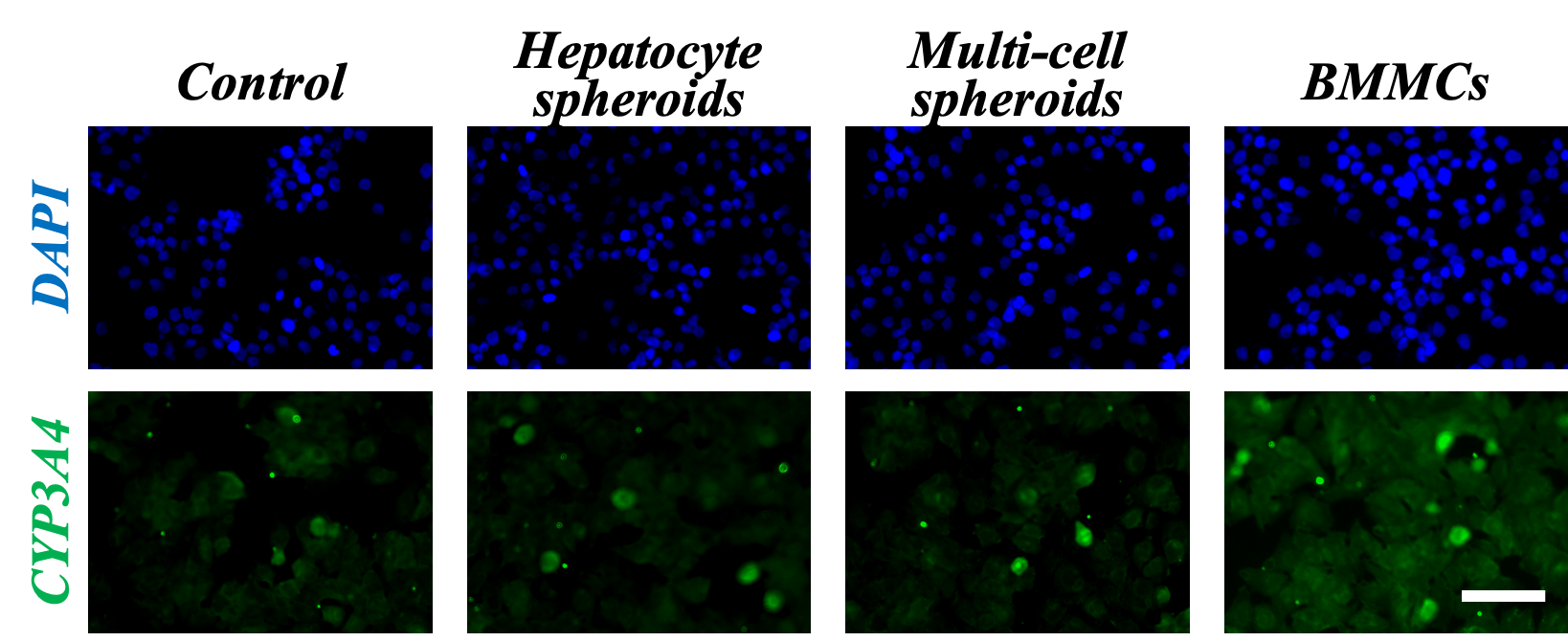


**Figure S11.** Single channel fluorescence staining images of DAPI and CYX3A4 from different groups. Scale bar is 100 μm.


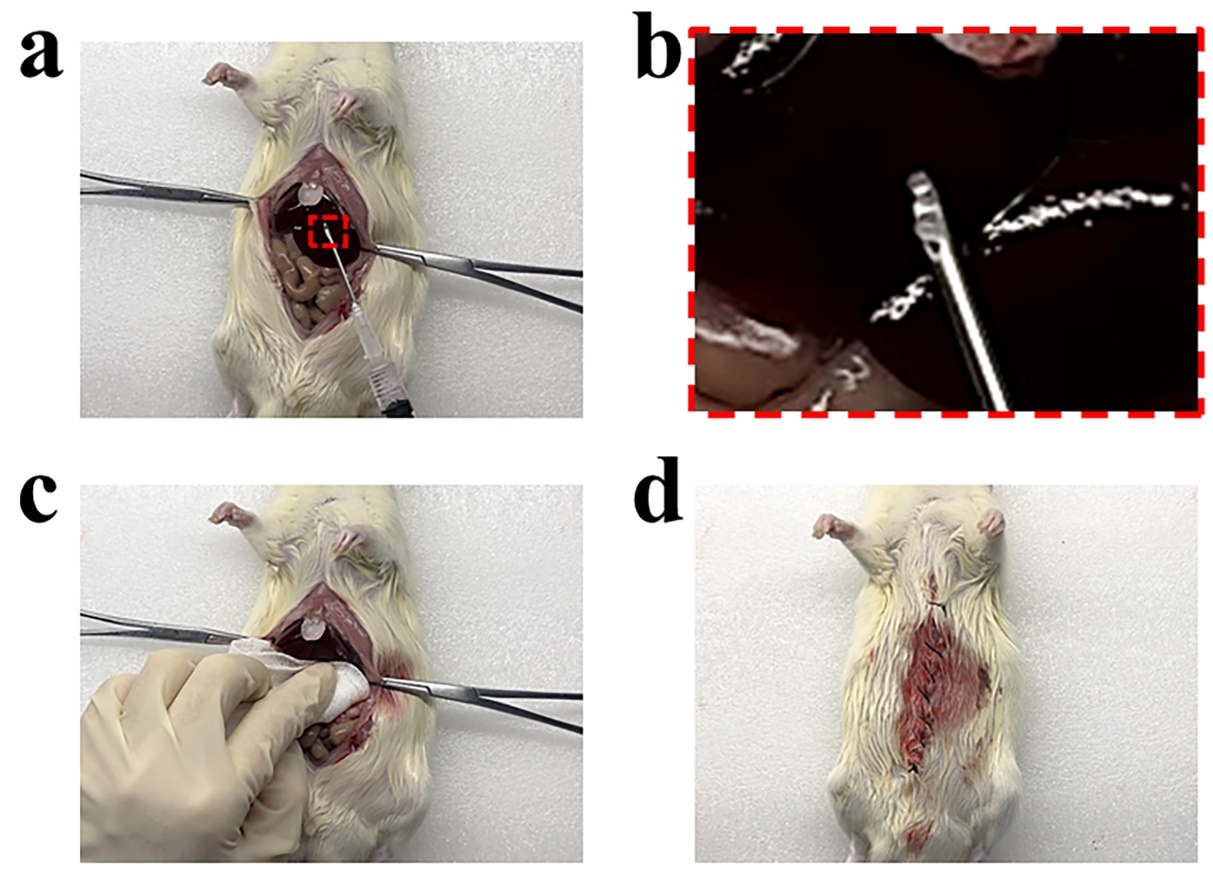


**Figure S12.** Photograph of the animal experiment process. (a) In situ injection of biomimetic microparticles. (b) Enlarge photograph of the needle. (c) Apply pressure to stop bleeding after transplantation. (d) Suture.


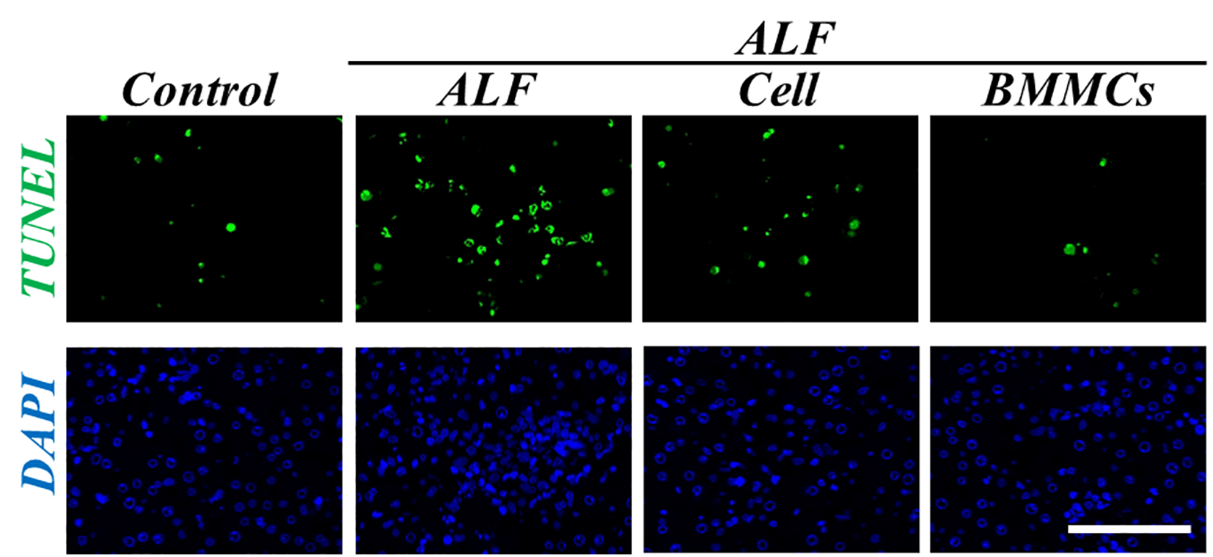


**Figure S13.** Single channel fluorescence staining images of TUNEL and DAPI from different groups. Scale bar is 100 μm.
